# Genomic surveillance and evolutionary dynamics of respiratory syncytial virus circulating in Tunisia post–COVID-19 pandemic lockdown restrictions

**DOI:** 10.1016/j.ijregi.2025.100609

**Published:** 2025-02-24

**Authors:** Greta Romano, Zoubeir Bouslah, Salma Abid, Francesca Di Giallonardo, Guglielmo Ferrari, Antonio Piralla, Awatef El Moussi, Yasmine Koumi, Latifa Charaa, Ichrak Landolsi, Hakim El Ghord, Valentina Curini, Maurilia Marcacci, Hanen Smaoui, Ahlem Gzara, Chekib Zedini, Alessandro Ripani, Wafa Achour, Khaled Mnif, Cesare Cammà, Alessio Lorusso, Ilhem Boutiba Ben Boubaker

**Affiliations:** 1 Fondazione IRCCS Policlinico San Matteo, Microbiology and Virology Department, Pavia, Italy; 2 University of Tunis ElManar, Faculty of Medicine of Tunis, Tunis, Tunisia; 3 Charles Nicolle Hospital, Laboratory of Microbiology, National Influenza Centre, National Reference Lab of Respiratory Viruses, Tunis, Tunisia; 4 The Kirby Institute, The University of New South Wales (UNSW), Sydney, Australia; 5 Béchir Hamza Children's Hospital, Pediatric intensive care unit, Tunis, Tunisia; 6 University Tunis El Manar, Faculty of Medicine of Tunis, Tunis, Tunisia; 7 Ministry of Health, Primary Health Care Directorate, Tunisia; 8 Istituto Zooprofilattico Sperimentale dell'Abruzzo e del Molise, Teramo, Italy; 9 Béchir Hamza Children's Hospital, Laboratory of Microbiology, Tunis, Tunisia; 10 National Bone Marrow Transplant Center, Microbiology Laboratory, Tunis, Tunisia

**Keywords:** Human respiratory syncytial virus (HRSV), Whole genome sequencing (WGS), Genetic diversity, Tunisia, Surveillance

## Abstract

**Objectives:**

Human respiratory syncytial virus (hRSV) is the leading cause of severe respiratory infections in children worldwide. Severe cases often require intensive care. Respiratory syncytial virus (RSV) is classified into two main antigenic groups: RSV-A and RSV-B. Recent molecular advancements have significantly enhanced our ability to control and understand RSV infections. This study investigated the epidemiologic and genetic characteristics of the RSV in Tunisia, focusing on its evolutionary dynamics.

**Methods:**

Between October and December 2021, 92 samples were collected from Tunisian patients hospitalized for mild-to-severe acute respiratory infections. Laboratory analyses, including real-time reverse transcription-polymerase chain reaction and whole genome sequencing (WGS), were performed to identify and characterize the RSV strains. Phylogenetic analyses were performed to compare the Tunisian sequences with the global RSV sequences from 2012 to 2022.

**Results:**

Of the 92 patients (mean age 1 year and 6 months), 96.9% of the samples tested positive for RSV-A, and 22 samples exhibited co-infections with other respiratory viruses. WGS was successfully performed on the 74 samples. Phylogenetic analysis identified six distinct clades of RSV-A circulating in Tunisia, indicating multiple parallel introductions of the virus into the country. Specific Tunisian clades showed genetic similarities to RSV strains from Argentina, Belgium, and the Philippines.

**Conclusions:**

This study underscores the genetic diversity of RSV in Tunisia, with multiple introductions after the lifting of COVID-19 restrictions in 2021. WGS revealed significant genetic heterogeneity within RSV-A, which could affect the effectiveness of vaccines and therapeutic strategies. Continued surveillance, particularly, in resource-limited regions, is crucial to inform and guide future interventions.

## Highlights

• Human respiratory syncytial virus (hRSV)-A genetic diversity revealed multiple clades in Tunisia during 2021-2022.
• Whole genome sequencing identified 73 hRSV-A strains with high genetic variability.
• Post–COVID-19 lockdowns led to a resurgence of hRSV and unique viral introductions.
• Coinfections with Enterovirus and Alphacoronavirus were detected in 11.7% of cases.
• Genomic surveillance is essential to monitor hRSV evolution and guide vaccine strategies.

## Introduction

Human respiratory syncytial virus (hRSV) is the most common cause of respiratory viral infections during early childhood. Respiratory syncytial virus (RSV) is a negative-sense, single-stranded, non-segmented RNA virus belonging to the family *Paramyxoviridae* the subfamily *Pneumovirinae* [1]. The RSV genome is 15.2 kilobases long and contains 10 genes that encode non-structural and structural proteins [2]. Of the structural proteins, the attachment glycoprotein (G) and fusion glycoprotein (F) are critical for initiating infection and serve as targets for neutralizing antibodies [2].

RSV is associated with a broad spectrum of illnesses, ranging from mild symptoms similar to those of the common cold to more severe conditions including life-threatening croup, bronchiolitis, and pneumonia. Severe cases may require admission to the pediatric intensive care unit, intubation, and mechanical ventilation [3]. Bronchiolitis caused by RSV is often more severe and prolonged than that caused by other viral agents, such as rhinovirus, human metapneumovirus, or parainfluenza virus [4]. Consequently, the accurate diagnosis and appropriate treatment of RSV infections are of particular importance.

Worldwide, approximately 34 million new pediatric cases of RSV-associated lower airway disease occur annually, resulting in an estimated 200,000 deaths, most of which occur in low-income countries with limited access to standardized health care [5]. The importance of detecting RSV in infants with bronchiolitis is emphasized by the observation that the severity and progression of the disease do not differ significantly when multiple respiratory viruses are found simultaneously with RSV compared with cases where RSV is the sole cause of infection [6,7].

RSV exhibits significant viral diversity, with two major antigenic groups, RSV-A and RSV-B, circulating worldwide, each composed of multiple subgenotypes [4]. Modern diagnostic methods for identifying RSV in lower airway samples include molecular techniques such as real-time reverse transcription-polymerase chain reaction (RT-PCR) for species classification (A or B), sequencing methods (e.g. Sanger sequencing and next-generation sequencing) for typing (F and G proteins), and recovery of whole genome sequences (WGS) [8].

Since 2023, one RSV vaccine for pregnant women at 32-36 weeks gestation and a monoclonal antibody for infants born or entering their first RSV season have been approved by regulatory authorities, marking a significant advancement in the prevention and management of this virus [9,10].

However, there is an increasing need for detailed and continuous surveillance of RSV in resource-rich and -limited settings worldwide [11].

During the COVID-19 pandemic, Tunisia implemented a series of lockdowns and restrictive measures to curb the spread of the virus. The first nationwide lockdown was announced on March 22, 2020, followed by subsequent restrictions, including a general lockdown in May 2021, as the number of cases surged. The easing of these restrictions in 2021 led to the resurgence of respiratory viruses, including RSV, which were suppressed during the pandemic [12].

In this study, 73 RSV-A–positive samples collected between October and December 2021 from children's hospitals in Tunisia were analyzed using WGS and targeted sequencing of G proteins. Sequencing efforts were designed to investigate the epidemiologic and evolutionary dynamics of RSV in Tunisia using viral genomic data, providing critical insights into the identification of RSV strains that contribute to global circulation.

## Materials and methods

### Study population, diagnostic analysis, and WGS

Between October and December 2021, a total of 92 nasopharyngeal swabs were collected from patients across various governorates of Tunisia (Supplementary Table 1). The data set included 54 males and 40 females, with a mean age of 608 days (approximately 1 year and 6 months; age range 1 day to 68 years). The majority of patients were children hospitalized in intensive care units for severe acute respiratory infections at the Children's Hospital of Tunis (n = 86) and the National Bone Marrow Transplant Center (n = 3), whereas five were outpatients presenting with influenza-like illness.

Total RNA was extracted from the nasopharyngeal swabs and tested for the presence of the RSV genome using the RealStar RSV RT-PCR Kit 3.0 (Altona Diagnostics GmbH, Hamburg, Germany).

RSV-positive samples were selected for WGS using the sequence-independent single-primer amplification protocol [[13], [14], [15]]. PCR products were purified using the Expin PCR SV kit (GeneAll Biotechnology Co., Seoul, Korea) and quantified using the Qubit DNA HS Assay Kit (Thermo Fisher Scientific, Waltham, MA, USA). Library preparation was performed using the Illumina DNA Prep (M) Tagmentation kit (96 Samples) (Illumina Inc., San Diego, CA, USA), following the manufacturer's protocol.

Deep sequencing was conducted on the NextSeq500 platform (Illumina Inc., San Diego, CA, USA) using the NextSeq 500/550 Mid Output Reagent Cartridge v2 (300 cycles) to generate standard 150-bp paired-end reads. Quality checks and trimming of the raw read data were performed using FastQC v0.11.5 and Trimmomatic v0.36, respectively. The resulting FASTQ files were uploaded to CZ-ID (https://czid.org/) to identify the closest matching RSV sequences in GenBank. The best matching sequence was used as a reference for mapping via iVar v1.3.1 (intrahost variant analysis of replicates; github.com/andersen-lab/ivar) to generate consensus sequences.

Consensus sequences with horizontal coverage greater than 50% were included in the phylogenetic analysis.

### Phylogenetic analysis

Two data sets were prepared for analysis: one included full-length RSV-A sequences retrieved from the Global Initiative on Sharing All Influenza Data (GISAID) database (https://gisaid.org/) spanning from January 2012 to June 30, 2022, and the other contained G gene sequences downloaded from National Center for Biotechnology Information (https://www.ncbi.nlm.nih.gov/nucleotide/) [16]. All FASTA files were aligned using MAFFT v7.490 [17] in Geneious (https://www.geneious.com). Sequences that were very partial (not covering the G gene) or lacked sampling dates were excluded from analysis.

**Figure 1:**
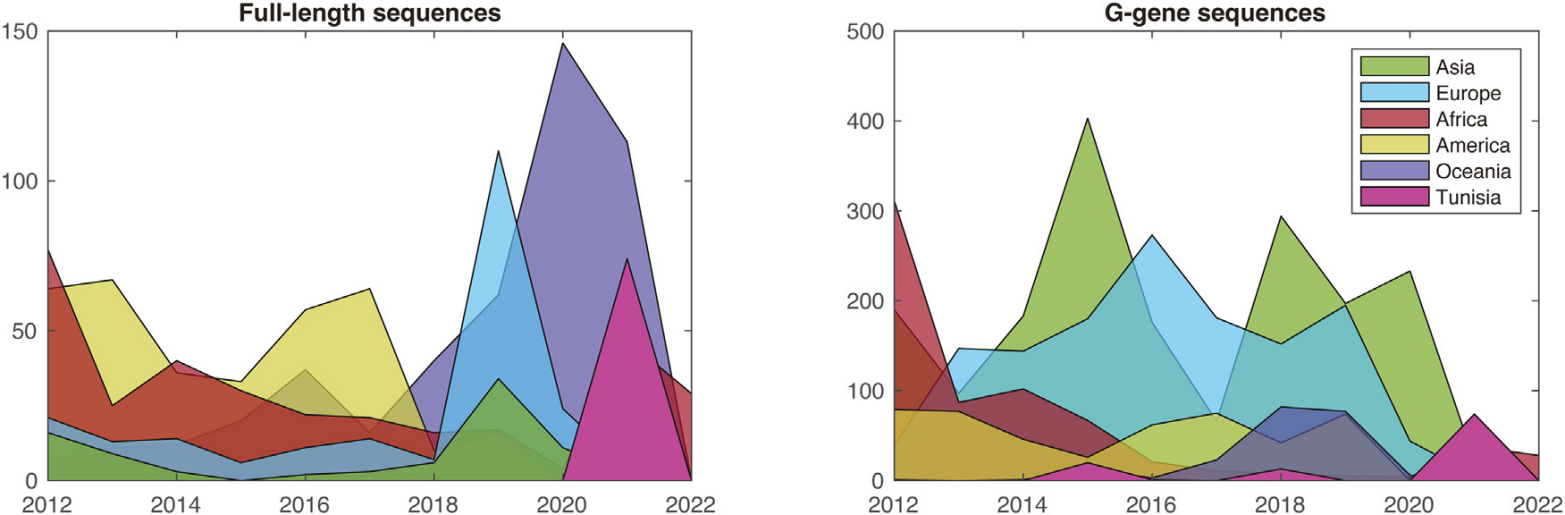
Distribution of the sequence data per country. The number of sequences collected between 2012 and 2022 and used in this study is displayed for the full-length data (left panel) and G gene data (right panel), with each segment color-coded by country of origin: green represents Asia, cyan represents Europe, red represents Africa, yellow represents the Americas, purple represents Oceania, and pink represents Tunisia.

The initial global data sets comprised 1537 full-length RSV-A genomes (including 73 sequences generated in the present study) and 4710 G gene sequences (including 56 sequences from the present study) (Figure 1). A maximum likelihood tree was constructed using FastTree v2.1.11 [18] in Geneious. To simplify the visualization of phylogenies, global data sets were subsampled for full-length and G gene data. Random subsets of sequences were extracted and new maximum likelihood trees were constructed using IQ-TREE v1.6.12, with integrated model selection and ultrafast bootstrap approximation [19].

### Genome mutation analysis

A multiple alignment of the G protein from 56 Tunisian RSV was derived using MG642035.1 as a reference. A subset of G protein sequences was selected using a cutoff based on informative nucleotides (<5% of N nucleotides). The Snipit pipeline (https://github.com/aineniamh/snipit) [20] was used to assess the presence of conserved non-synonymous mutations among the Tunisian RSV strains. The tool was modified to select non-synonymous mutations and work with amino acid notations.

## Results

### Comprehensive genomic analysis of the Tunisian RSV-A and RSV-B strains: high sequencing coverage and coinfections with other respiratory viruses

Of the 92 samples tested using the RealStar RSV RT-PCR Kit 3.0 (Altona Diagnostics GmbH, Hamburg, Germany), 88 tested positive for RSV-A, with a mean cycle threshold value of 20.92 (range: 13.4-30.5), and two tested positive for RSV-B, with cycle threshold values of 21.4 and 29.0, respectively (Supplementary Table 1) . WGS was successfully performed on 74 of the 90 positive samples (73 for RSV-A and one for RSV-B).

Mapping analysis was conducted using the reference RSV-A isolates OX02-9571-V01 (complete genome, MZ515567.1) and NC_001781.1 for RSV-B. The median number of raw reads obtained per sample was 4,439,725.87 (range: 24,388-41,660,064), whereas the median number of mapped reads was 104,949 (range: 0-1,374,021). As a result, 73 nearly complete consensus sequences were obtained for the Tunisian RSV-A strains and one for RSV-B. Of these sequences, the median vertical coverage (Vcov) was 992.56× (range: 4-6677.9×), and the horizontal coverage (Hcov) was 94% (range: 52-98%). The mean consensus sequence length for the RSV-A samples was 14,826 nucleotides (range: 13,336-15,094 nucleotides).

Metagenomic analysis performed using the CZ-ID platform identified coinfections with other respiratory viruses in 13 of the 90 (11.7%) samples. Reads were assigned to various species, including members of the Enterovirus genus (*E. cerhino, E. alpharhino, E. alphacoxsachiae, E. deconjuncti*), *Human coronavirus 229E* (HCoV-229E), and *Orthorubulavirus hominis* (Supplementary Table 1). Nearly complete consensus sequences were obtained for HCoV-229E (Hcov: 99.8%; Vcov: 961.5×), *E. alphacoxsachiae* (Hcov: 83%; Vcov: 903.3×), and *E. cerhino* (Hcov: 96%; Vcov: 70.4×).

### Phylogenetic insights into the global and local evolutionary dynamics of respiratory syncytial virus-A: evidence of multiple introductions and unique clades in Tunisia

**Figure 2:**
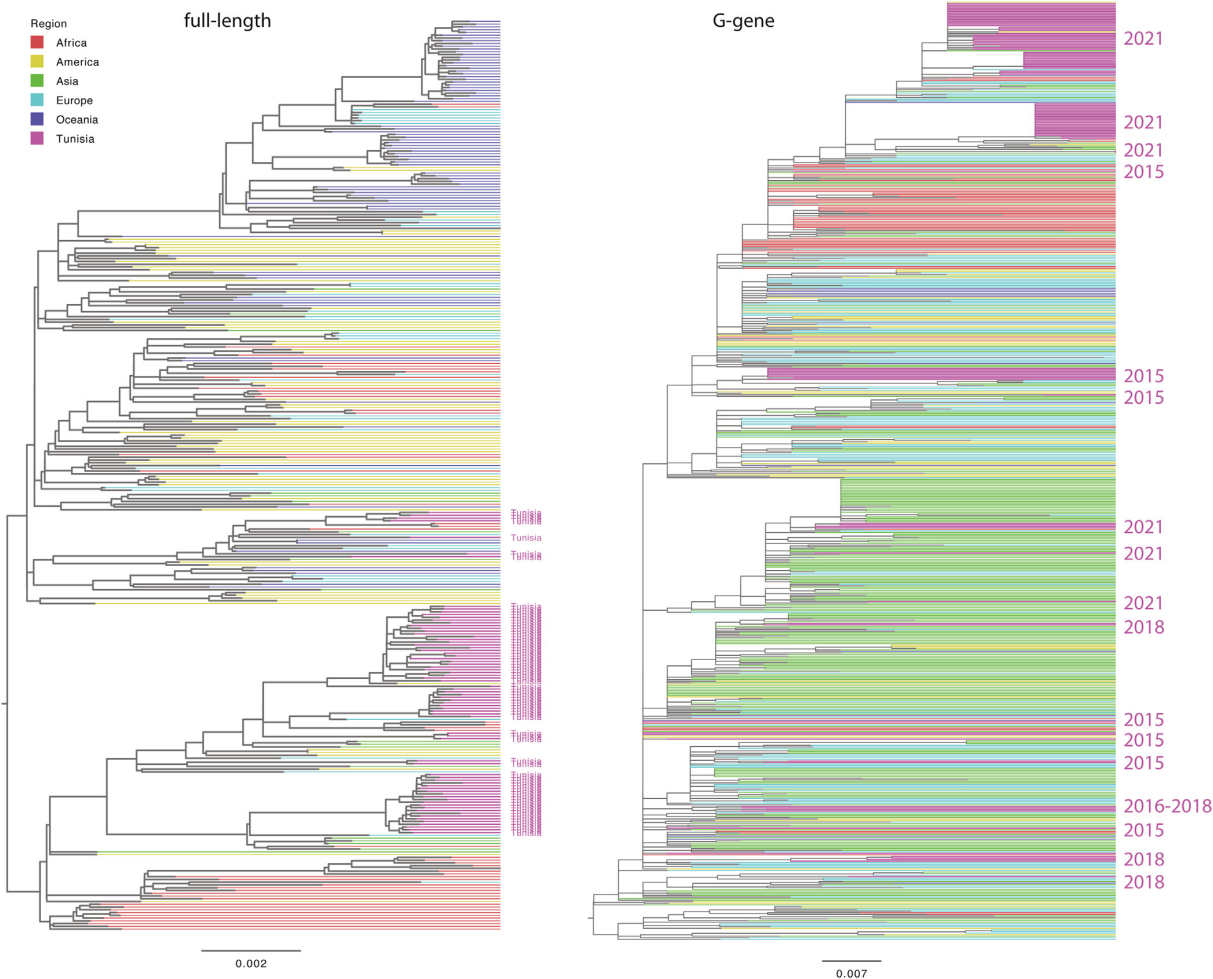
Global phylogeny. The maximum likelihood trees of the respiratory syncytial virus-A full-length sequences (left) and G gene sequences (right) are shown. Branches are colored based on the country of origin, with Tunisian sequences highlighted in pink. For G gene sequences, the sampling year for Tunisian sequences is annotated on the right-hand side. Branch lengths represent nucleotide substitutions per site.

**Figure 3:**
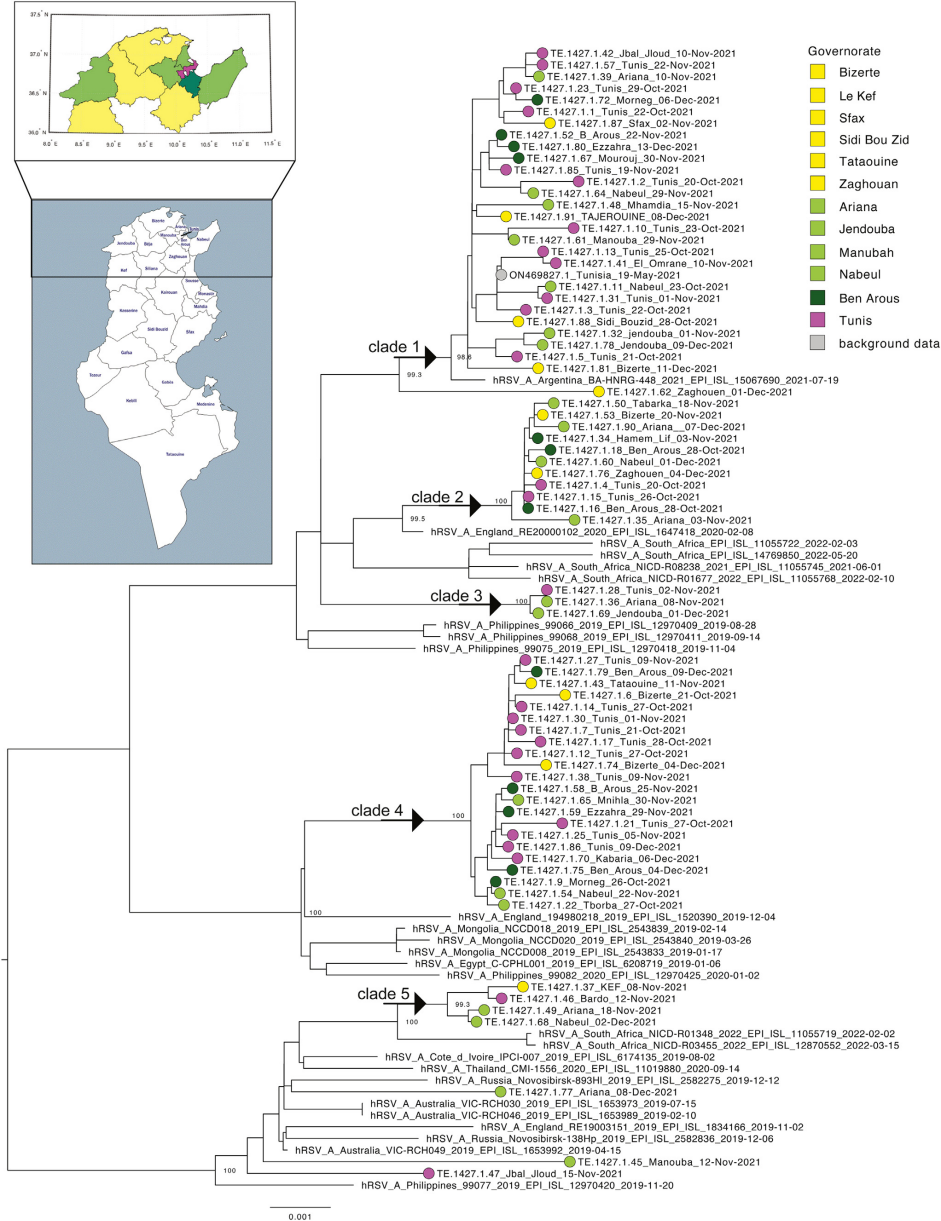
Phylogenetic tree of full-length strains from Tunisia. An extracted view of the tree from Figure 2 is shown, highlighting clades containing Tunisian data. The tips are color-coded by the Tunisian governorate and are annotated on the accompanying map. The five clades are indicated with arrows. Branch lengths represent nucleotide substitutions per site.

Phylogenetic analysis was conducted using the Tunisian RSV-A sequences obtained here, full-length RSV-A sequences retrieved from the GISAID database (from January 2012 to June 30, 2022), and G gene RSV-A sequences downloaded from the National Center for Biotechnology Information database [16]. Both data sets contained sequences from all over the world. For full-length sequences, the largest proportion of data was from Oceania (30.2%), followed by America (23.4%), Africa (21.3%), Europe (14.7%), Asia (5.6%), and Tunisia (4.8%). For G gene sequences, the largest proportion of data were from Asia (39.5%), followed by Europe (28.9%), Africa (14.5%), America (10.4%), Oceania (4.4%), and Tunisia (2.3%) (Figure 1). The Tunisian strains were scattered across the global phylogeny (G gene), indicating the widespread distribution of RSV-A strains in the country (Figure 2). Multiple introductions have been observed since 2015 when G protein sequences were available from Tunisian strains. Some clusters of circulation were observed in 2021, which were also supported by full-length sequence analysis. Multiple Tunisian-specific clades containing viral isolates from 2021 were identified, including three large clades, indicating numerous parallel introductions of RSV-A into the country (Figure 3). Within Tunisia, the virus isolates showed no geographic separation, and all clades contained a mix of virus sequences from Tunis and surrounding areas. There was no link between the isolates from Tunisia sampled in 2018 and those from 2021 (Figure 2). Thus, the latter virus variants are new introductions and not the re-emergence of circulating 2018 variants. Clade one was genetically close to the isolates from Argentina, whereas all other clades had no genetic link or a very distant genetic link to other isolates (Figure 3).

### Genetic variance of the G protein from RSV-A Tunisia

To select crucial amino acid mutation sites that may affect the virulence of the variants, the modified Snipit [20] script (https://github.com/aineniamh/snipit) was used to visualize the relative changes of each amino acid site compared with the reference sequence. A subset of 31 G protein sequences was selected using a cutoff based on informative nucleotides. Only consensus sequences with less than 5% non-informative nucleotides (N generic nucleotides) were targeted for analysis. The G protein of RSV-A contains three domains: the cytoplasmic domain (residues 1-37), the transmembrane domain (residues 38-66), and the ectodomain (residues 67-298) [21].

**Figure 4:**
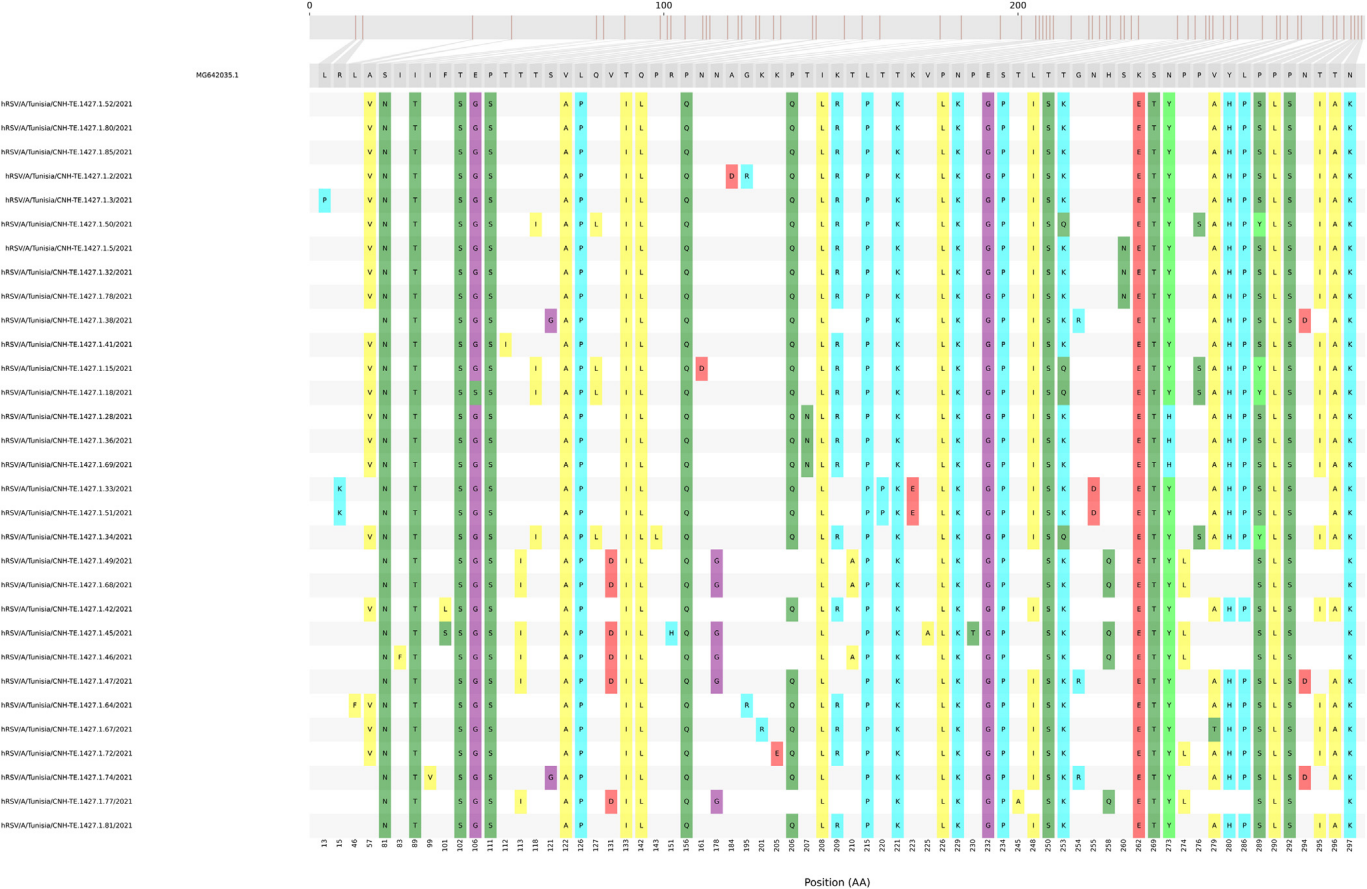
Analysis of the amino acid variance of the RSV-A sequences based on the G protein alignment. A total of 31 G protein sequences were selected based on the percentage of informative nucleotides (>95%) in the sequence. The figure was rendered using the *Snipit* tool with an improvement of the pipeline for amino acid visualization (https://github.com/aineniamh/snipit).

Comparing the G protein sequence alignments with the reference genome (MG642035.1, 1984), 69 of 298 (23%) amino acid positions showed at least one change (Figure 4).

Of these, 65 of 232 (28%) changes occurred within the ectodomain, whereas only two of 37 (5%) and two of 28 (7%) occurred within the cytoplasmic and transmembrane domains, respectively (Figure 4). The ectodomain G protein contains two hypervariable regions in the C-terminal region (residues 67-164 and 199-298) that present most of the mutations (60 of 196 residues, 31%) and are responsible for variability [21]. Finally, within the G protein ectodomain, there was a 13-amino acid sequence (164-176aa) that was highly conserved across all RSV strains [21] in which no mutations were observed (Figure 4).

The mutations observed in the hypervariable regions of the 2021 Tunisian RSV-A strains were probably the main cause of the introduction of the latter virus variants rather than the re-emergence of circulating 2018 RSV-A variants.

## Discussion

RSV is one of the most common respiratory viruses that typically causes cold-like symptoms. Although most patients with RSV recover fully within a week without requiring medical assistance, the virus can lead to severe illness and even death in neonates (<6 months of age), the elderly (>65 years), and immunocompromised individuals. However, research on the incidence and transmission of RSV in Africa is limited.

In this study, WGS of RSV strains circulating in Tunisia was performed to evaluate their evolution. RSV was predominantly detected as a single infection, although coinfections were confirmed in 28.7% of cases. This study analyzed RSV-positive samples collected in Tunisia between 2021 and 2022. A significant resurgence of RSV was observed during this period after the lifting of COVID-19 restrictions, highlighting RSV as the leading cause of serious respiratory illness in early childhood. Similar post-pandemic surges in RSV cases were reported in several other countries in autumn of 2021 [[22], [23], [24]]. In Africa, limited reports have documented RSV circulation in the post–COVID-19 years, primarily, in South Africa and Senegal [25,26].

Phylogenetic analysis revealed that Tunisian RSV strains from 2021 to 2022 belonged to at least five distinct clades, suggesting multiple parallel introductions of RSV-A into the country. The G protein consensus analysis revealed the 28% of amino acid alteration in hypervariable sites of the sequence, with respect to the reference. These mutations in RSV-A strains are likely to be the primary reason for the introduction of new viral variants circulating in Tunisia in 2021.

This finding underscores the importance of expanding WGS efforts, particularly, in the context of introducing vaccines and monoclonal antibodies against RSV. Next-generation sequencing could play a pivotal role in enhancing the epidemiologic monitoring and tracking of virulent variants that may contribute to immune or therapeutic escape.

This study highlighted the significant impact of RSV on respiratory illnesses in Tunisia, particularly, in children. Coinfections with RSV and other respiratory viruses, such as *Enterovirus* and *Alphacoronavirus*, were observed in 11.7% of cases. However, the disease severity is primarily attributed to RSV, even in cases of co-infection. These findings emphasize the need for continuous genomic surveillance to monitor the genetic evolution of RSV infections in Tunisia.

The resurgence of RSV observed in Tunisia during 2021-2022 coincided with the lifting of the strict COVID-19 lockdown measures. The lockdown and associated social distancing likely suppressed the circulation of respiratory viruses, creating a population with reduced immunity owing to limited exposure. This phenomenon has been reported worldwide and is consistent with our findings of increased RSV activity after the pandemic [[27], [28], [29], [30], [31], [32]]. Understanding the impact of these restrictions is critical to anticipate shifts in viral epidemiology and guide public health strategies in similar scenarios.

In conclusion, this study revealed substantial genetic heterogeneity among RSV strains circulating in Tunisia in 2021. The post-pandemic period was marked by a resurgence of respiratory viruses, particularly, RSV, accompanied by increased genetic variability. Continued WGS is essential for tracking the evolution of the F protein, a key target for monoclonal antibody therapies, and guiding the development of effective interventions against RSV.

## Declarations of competing interest

The authors declare no conflict of interest. The mention of trade names or commercial products in this article is solely for the purpose of providing specific information and does not imply a recommendation or endorsement by the IZSAM.

## Appendix Supplementary materials

Image, application 1

Image, application 2

Image, application 3

## Footnotes

Supplementary material associated with this article can be found, in the online version, at doi:10.1016/j.ijregi.2025.100609.
