# Supplementary figures and images for: Genomic surveillance and evolutionary dynamics of respiratory syncytial virus circulating in Tunisia post–COVID-19 pandemic lockdown restrictions
Source: IJID Reg. 2025 Feb 24;15:100609. doi: 10.1016/j.ijregi.2025.100609 (PMC11987636; doi:10.1016/j.ijregi.2025.100609)

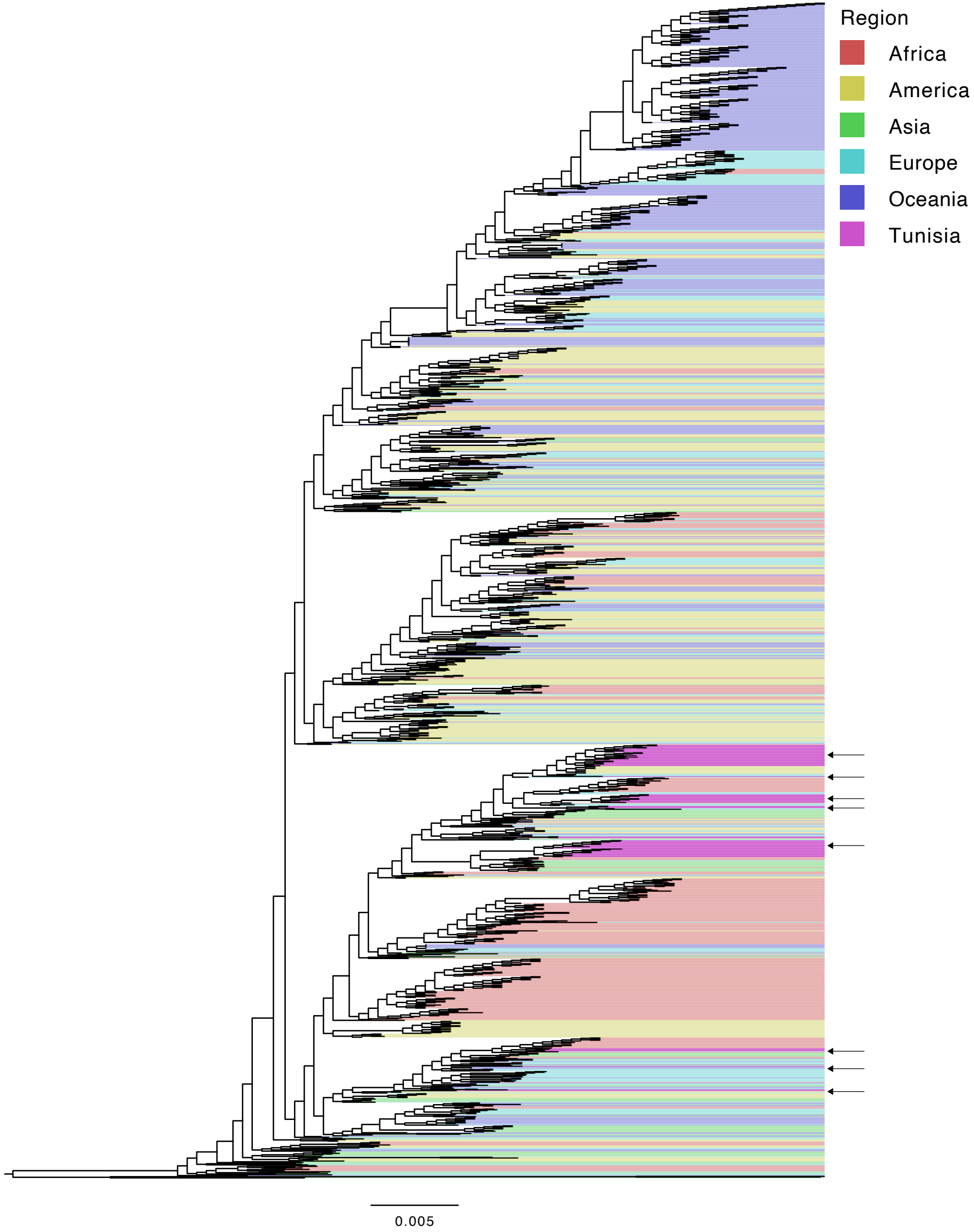

Supplement: Supplementary file 1 [file mmc1.pdf]

Region

- Africa
- America
- Asia
- Europe
- Oceania
- Tunisia

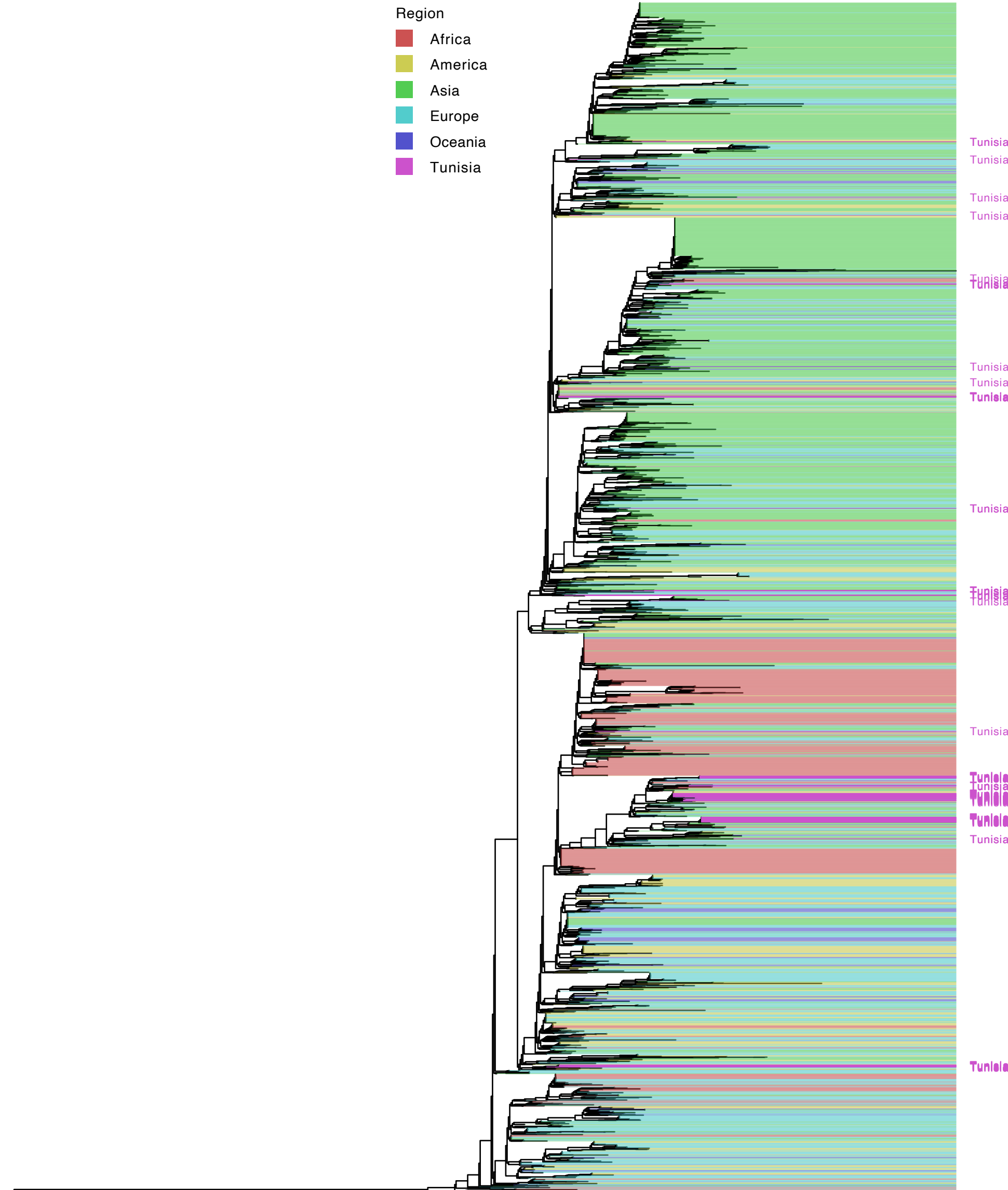

0.04

Supplement: Supplementary file 2 [file mmc2.pdf]
