# Supplementary material for: Genomic surveillance and evolutionary dynamics of respiratory syncytial virus circulating in Tunisia post–COVID-19 pandemic lockdown restrictions
Source: IJID Reg. 2025 Feb 24;15:100609. doi: 10.1016/j.ijregi.2025.100609 (PMC11987636; doi:10.1016/j.ijregi.2025.100609)
Supplement: Supplementary file 3 [file mmc3.docx]

Supplementary Table 1. Epidemiologic and diagnostic data of patients with respiratory infections.

| No. | Sampling date | Gender | Geographic origin | Age | Hospital | hRSV Ct | Other respiratory viruses detected |
| --- | --- | --- | --- | --- | --- | --- | --- |
| 1 | 2021-10-22 | Male | Tunis | 2 months | Intensive Care Unit - Children’s Hospital of Tunis | 16,54 |  |
| 2 | 2021-10-20 | Female | Tunis | 10 days | Intensive Care Unit – Children’s Hospital of Tunis | 21,09 |  |
| 3 | 2021-10-22 | Male | Tunis | 2.5 months | Intensive Care Unit – Children’s Hospital of Tunis | 17,87 |  |
| 4 | 2021-10-20 | Male | Tunis | 1 month | Intensive Care Unit – Children’s Hospital of Tunis | 17,49 |  |
| 5 | 2021-10-21 | Female | Tunis | 1 month | Intensive Care Unit – Children’s Hospital of Tunis | 22,39 |  |
| 6 | 2021-10-21 | Female | Bizerte | 1 month | Intensive Care Unit – Children’s Hospital of Tunis | 22,27 |  |
| 7 | 2021-10-21 | Female | Tunis | 2 months | Intensive Care Unit – Children’s Hospital of Tunis | 20,27 | 14 reads *Enterovirus cerhino* |
| 8 | 2021-10-26 | Male | Tunis | 3 months | Intensive Care Unit – Children’s Hospital of Tunis | 21,43 |  |
| 9 | 2021-10-26 | Male | Ben Arous | 1 month | Intensive Care Unit – Children’s Hospital of Tunis | 22,87 |  |
| 10 | 2021-10-23 | Male | Tunis | 1 month | Intensive Care Unit – Children’s Hospital of Tunis | 28,13 |  |
| 11 | 2021-10-23 | Male | Nabeul | 4 months | Intensive Care Unit – Children’s Hospital of Tunis | 23,63 |  |
| 12 | 2021-10-27 | Male | Tunis | 1 month | Intensive Care Unit – Children’s Hospital of Tunis | 21,41 |  |
| 13 | 2021-10-25 | Male | Tunis | 14 days | Intensive Care Unit – Children’s Hospital of Tunis | 18,45 |  |
| 14 | 2021-10-27 | Female | Tunis | 2 months | Intensive Care Unit – Children’s Hospital of Tunis | 25,29 |  |
| 15 | 2021-10-26 | Male | Tunis | 2 months | Intensive Care Unit – Children’s Hospital of Tunis | 18,37 |  |
| 16 | 2021-10-28 | Male | Ben Arous | 1 month | Intensive Care Unit – Children’s Hospital of Tunis | 20,70 | 4 reads hCov-229E |
| 17 | 2021-10-28 | Female | Tunis | 1 month | Intensive Care Unit – Children’s Hospital of Tunis | 19,13 |  |
| 18 | 2021-10-28 | Female | Ben Arous | 1 month | Intensive Care Unit – Children’s Hospital of Tunis | 19,27 |  |
| 19 | 2021-10-28 | Male | Tunis | 25 days | Intensive Care Unit – Children’s Hospital of Tunis | 18,84 |  |
| 20 | 2021-10-27 | Male | Tunis | 1 month | Intensive Care Unit – Children’s Hospital of Tunis | 21,64 |  |
| 21 | 2021-10-27 | Male | Tunis | 1 month | Intensive Care Unit – Children’s Hospital of Tunis | 17,69 |  |
| 22 | 2021-10-27 | Male | Manouba | 2 months | Intensive Care Unit – Children’s Hospital of Tunis | 20,08 |  |
| 23 | 2021-10-29 | Male | Tunis | 1 month | Intensive Care Unit – Children’s Hospital of Tunis | 15,59 |  |
| 24 | 2021-11-06 | Female | Manouba | 21 days | Intensive Care Unit – Children’s Hospital of Tunis | 30,05 |  |
| 25 | 2021-11-05 | Male | Tunis | 2 weeks | Intensive Care Unit – Children’s Hospital of Tunis | 20,89 |  |
| 26 | 2021-11-02 | Female | Tunis | 1 month | Intensive Care Unit – Children’s Hospital of Tunis | 25,58 | 331 reads *Enterovirus cerhino* |
| 27 | 2021-11-09 | Female | Tunis | 2 months | Intensive Care Unit - Children’s Hospital of Tunis | 18,44 |  |
| 28 | 2021-11-02 | Male | Tunis | 1 month | Intensive Care Unit – Children’s Hospital of Tunis | 17,87 |  |
| 29 | 2021-11-01 | Male | Tunis | 2 weeks | Intensive Care Unit – Children’s Hospital of Tunis | 17,81 |  |
| 30 | 2021-11-01 | Male | Tunis | 2 days | Intensive Care Unit – Children’s Hospital of Tunis | 15,77 |  |
| 31 | 2021-11-01 | Male | Tunis | 2 months | Intensive Care Unit – Children’s Hospital of Tunis | 17,32 |  |
| 32 | 2021-11-01 | Female | Jendouba | 2 months | Intensive Care Unit – Children’s Hospital of Tunis | 21,77 |  |
| 33 | 2021-11-03 | Male | Tunis | 2 weeks | Intensive Care Unit – Children’s Hospital of Tunis | 16,66 |  |
| 34 | 2021-11-03 | Female | Ben Arous | 3 months | Intensive Care Unit – Children’s Hospital of Tunis | 25,39 |  |
| 35 | 2021-11-03 | Female | Ariana | 5 months | Intensive Care Unit – Children’s Hospital of Tunis | 19,10 |  |
| 36 | 2021-11-08 | Female | Ariana | 3 weeks | Intensive Care Unit – Children’s Hospital of Tunis | 19,53 |  |
| 37 | 2021-11-08 | Male | Kef | 5 months | Intensive Care Unit – Children’s Hospital of Tunis | 23,86 | 30 reads *Enterovirus alpharhino* |
| 38 | 2021-11-09 | Female | Tunis | 3 weeks | Intensive Care Unit – Children’s Hospital of Tunis | 15,56 |  |
| 39 | 2021-11-10 | Female | Ariana | 4 months | Intensive Care Unit – Children’s Hospital of Tunis | 22,31 |  |
| 40 | 2021-11-10 | Female | Manouba | 1 month | Intensive Care Unit – Children’s Hospital of Tunis | 21,40^a^ |  |
| 41 | 2021-11-10 | Male | Tunis | 2 months | Intensive Care Unit – Children’s Hospital of Tunis | 19,19 |  |
| 42 | 2021-11-10 | Female | Ben Arous | 1 month | Intensive Care Unit – Children’s Hospital of Tunis | 18,28 |  |
| 43 | 2021-11-11 | Female | Tataouine | 2 months | Intensive Care Unit – Children’s Hospital of Tunis | 20,82 | 220 reads *Enterovirus alphacoxsackie* |
| 44 | 2021-11-16 | Female | Tunis | 3 months | Intensive Care Unit – Children’s Hospital of Tunis | 24,23 |  |
| 45 | 2021-11-12 | Male | Manouba | 2 months | Intensive Care Unit – Children’s Hospital of Tunis | 23,10 |  |
| 46 | 2021-11-12 | Female | Tunis | 3 weeks | Intensive Care Unit – Children’s Hospital of Tunis | 18,03 |  |
| 47 | 2021-11-15 | Female | Ben Arous | 2 weeks | Intensive Care Unit – Children’s Hospital of Tunis | 14,95 |  |
| 48 | 2021-11-15 | Male | Ben Arous | 1 month | Intensive Care Unit – Children’s Hospital of Tunis | 19,37 |  |
| 49 | 2021-11-18 | Female | Ariana | 3 months | Intensive Care Unit – Children’s Hospital of Tunis | 24,19 | 2 reads *Enterovirus cerhino* |
| 50 | 2021-11-18 | Male | Jendouba | 1 month | Intensive Care Unit – Children’s Hospital of Tunis | 20,28 |  |
| 51 | 2021-11-17 | Female | Ben Arous | 2 weeks | Intensive Care Unit – Children’s Hospital of Tunis | 18,28 |  |
| 52 | 2021-11-22 | Male | Ben Arous | 1 month | Intensive Care Unit – Children’s Hospital of Tunis | 15,51 |  |
| 53 | 2021-11-20 | Male | Bizerte | 2 months | Intensive Care Unit – Children’s Hospital of Tunis | 21,72 | 98 reads *Enterovirus cerhino* |
| 54 | 2021-11-22 | Female | Nabeul | 3 months | Intensive Care Unit – Children’s Hospital of Tunis | Negative |  |
| 55 | 2021-11-21 | Male | Jendouba | 2 months | Intensive Care Unit – Children’s Hospital of Tunis | 26,89 |  |
| 56 | 2021-11-21 | Male | Tunis | 1 month | Intensive Care Unit – Children’s Hospital of Tunis | 26,76 |  |
| 57 | 2021-11-22 | Male | Tunis | 2 months | Intensive Care Unit – Children’s Hospital of Tunis | 17,72 |  |
| 58 | 2021-11-25 | Male | Ben Arous | 1 month | Intensive Care Unit – Children’s Hospital of Tunis | 16,21 |  |
| 59 | 2021-11-29 | Female | Ben Arous | 1 week | Intensive Care Unit – Children’s Hospital of Tunis | 19,30 |  |
| 60 | 2021-12-01 | Female | Nabeul | 25 days | Intensive Care Unit – Children’s Hospital of Tunis | 19,81 |  |
| 61 | 2021-11-29 | Female | Manouba | 1 year | Intensive Care Unit – Children’s Hospital of Tunis | 16,18 |  |
| 62 | 2021-12-01 | Male | Zaghouan | 1 month | Intensive Care Unit – Children’s Hospital of Tunis | 24,77 |  |
| 63 | 2021-12-29 | Female | Ariana | 1 month | Intensive Care Unit – Children’s Hospital of Tunis | 26,11 |  |
| 64 | 2021-11-29 | Male | Nabeul | 20 days | Intensive Care Unit – Children’s Hospital of Tunis | 20,78 |  |
| 65 | 2021-11-30 | Male | Ariana | 2 months | Intensive Care Unit – Children’s Hospital of Tunis | 17,81 |  |
| 66 | 2021-11-30 | Male | Tunis | 1 month | Intensive Care Unit – Children’s Hospital of Tunis | Negative |  |
| 67 | 2021-11-30 | Female | Ben Arous | 1 month | Intensive Care Unit – Children’s Hospital of Tunis | 19,23 |  |
| 68 | 2021-12-02 | Male | Nabeul | 2 months | Intensive Care Unit – Children’s Hospital of Tunis | 20,45 | 7.163 reads *Enterovirus cerhino* |
| 69 | 2021-12-01 | Female | Jendouba | 2 months | Intensive Care Unit – Children’s Hospital of Tunis | 21,60 | 2 reads HCoV-229E |
| 70 | 2021-12-06 | Male | Tunis | 1 month | Intensive Care Unit – Children’s Hospital of Tunis | 23,91 |  |
| 71 | 2021-12-06 | Female | Béja | 1 year | Intensive Care Unit – Children’s Hospital of Tunis | 29,58 | 2 reads hCov-229E, 10 reads *Enterovirus alphacoxsackie*,  110 reads *Othorubulavirus hominis* |
| 72 | 2021-12-06 | Male | Ben Arous | 2 months | Intensive Care Unit – Children’s Hospital of Tunis | 20,83 |  |
| 73 | 2021-12-04 | Female | Tunis | 2 months | Intensive Care Unit – Children’s Hospital of Tunis | 25,72 |  |
| 74 | 2021-12-04 | Male | Bizerte | 2 months | Intensive Care Unit – Children’s Hospital of Tunis | 19,08 |  |
| 75 | 2021-12-04 | Male | Ben Arous | 3 months | Intensive Care Unit – Children’s Hospital of Tunis | 18,63 |  |
| 76 | 2021-12-04 | Female | Zaghouan | 15 days | Intensive Care Unit – Children’s Hospital of Tunis | 24,49 |  |
| 77 | 2021-12-08 | Male | Ariana | 20 days | Intensive Care Unit – Children’s Hospital of Tunis | 23,53 |  |
| 78 | 2021-12-09 | Male | Jendouba | 2 months | Intensive Care Unit – Children’s Hospital of Tunis | 19,66 |  |
| 79 | 2021-12-09 | Male | Ben Arous | 2 months | Intensive Care Unit – Children’s Hospital of Tunis | 21,53 | 423.646 reads HCoV-229E |
| 80 | 2021-12-13 | Male | Ben Arous | 14 days | Intensive Care Unit – Children’s Hospital of Tunis | 13,35 |  |
| 81 | 2021-12-11 | Female | Bizerte | 4 months | Intensive Care Unit – Children’s Hospital of Tunis | 15,47 |  |
| 82 | 2021-12-11 | Female | Ben Arous | 2 months | Intensive Care Unit – Children’s Hospital of Tunis | 29,00^a^ |  |
| 83 | 2021-12-11 | Male | Nabeul | 1 month | Intensive Care Unit – Children’s Hospital of Tunis | 26,92 |  |
| 84 | 2021-12-13 | Male | Nabeul | 20 days | Intensive Care Unit – Children’s Hospital of Tunis | 27,03 |  |
| 85 | 2021-11-19 | Male | Tunis | 1 month | Intensive Care Unit – Children’s Hospital of Tunis | 20,09 |  |
| 86 | 2021-12-09 | Male | Tunis | 1 month | Intensive Care Unit – Children’s Hospital of Tunis | 17,93 |  |
| 87 | 2021-11-02 | Male | Sfax | 68 years | University Hospital Center Hedi Chaker of Sfax | 23,50 |  |
| 88 | 2021-10-28 | Female | Sidi Bouzid | 7 months | Basic Health Center of Sidi Bouzid | 22,55 | 2 reads *Enterovirus alphacoxsackie* |
| 89 | 2021-10-28 | Male | Sidi Bouzid | 7 months | Basic Health Center of Sidi Bouzid | 28,91 |  |
| 90 | 2021-12-07 | Male | Ariana | 63 years | Basic Health Center of Ariana | 25,23 |  |
| 91 | 2021-12-08 | Male | Kef | 11 years | Tajerouine hospital | 24,76 | 2 reads *Enterovirus cerhino*, 18 reads *Enterovirus deconjuncti* |
| 92 | 2021-06-01 | Female | Tunis | 2 months | National Bone Marrow Transplant Center | 18,41 |  |

^a^Positive for hRSV-B.
